# Macrophages and osteoclasts: similarity and divergence between bone phagocytes

**DOI:** 10.3389/fimmu.2025.1683872

**Published:** 2025-10-08

**Authors:** Julia Halper, Bastien Dolfi, Stoyan Ivanov, Maria-Bernadette Madel, Claudine Blin-Wakkach

**Affiliations:** Université Côte d’Azur, CNRS, LP2M, Nice, France

**Keywords:** macrophages, osteoclasts, bone phagocytes, bone formation, remodeling, bone pathologies, bone healing, fusion capacity

## Abstract

Both macrophages and osteoclasts are vital immune components of the bone microenvironment. While macrophages play an essential role in phagocytosis, pathogen clearance and tissue remodeling, osteoclasts are well described for their bone resorption capacity. However, osteoclasts are much more than bone resorbing cells. While macrophages have been intensively studied as immune cells, the immune function of osteoclasts has long been neglected until recent evidence demonstrated that they play an important role in modulating immune responses. Both macrophages and osteoclasts exhibit the phenotypic and functional characteristic plasticity of the myeloid lineage, which depends on their origin and environment. Besides their common progenitors, osteoclasts and macrophages share several joint mechanisms ranging from cell fusion and phagocytosis to immune function and tissue remodeling. In this review, we discuss and illustrate the functional and characteristic parallels between macrophages and osteoclasts.

## Highlights

- Osteoclasts and macrophages are both important regulators of bone formation and remodeling. They are also key players in bone pathologies and bone healing.
- They share many functional and phenotypical properties, including a fusion capacity. However, only osteoclasts are physiologically multinucleated while macrophages fusion is associated with pathological conditions
- BM macrophages and osteoclasts remain poorly explored for their origin, diversity and function compared to other tissue macrophages. In particular, osteoclast diversity and immune function have been neglected until recently.
- The finding that both osteoclasts and macrophages contribute to immune responses demonstrates that the role of OCLs extends far beyond their bone resorption activity and expanded the scope of osteoimmunology

## Introduction

Macrophages (Mφ) and osteoclasts (OCLs) both arise from the hematopoietic lineage and belong to the monocytic family. Monocytic cells are characterized by their ability to recognize and respond to danger signals (e.g. infection and tissue damage) and by their phagocytic properties and high cellular plasticity (1, 2). Besides their common origin, there are many parallels between Mφs and OCLs, one of the main being a common tissue degrading and phagocytic function. Like Mφs in tissues, OCLs are essential for maintaining bone homeostasis. Bone is constantly remodeled, while maintaining the balance between bone formation and resorption through osteoblasts and OCLs, respectively (3). This is a highly dynamic process in which OCLs degrade the bone matrix through the release of protons and catalytic enzymes. Bone resorption by OCLs is an important and necessary physiological process for bone growth, fracture healing as well as tooth eruption and maintenance of adequate blood calcium levels. In a subsequent step, new bone is formed by osteoblasts, which are in turn supported by marrow-resident Mϕ. However, pathological conditions such as estrogen deficiency or traumatic fracture events promote abnormal OCL differentiation resulting in accelerated bone resorption as well as changes in local Mϕ function.

While Mφs and OCLs are both myeloid cells, the role of OCLs as innate immune cells has long been neglected. Similar to Mφs, and in addition to their resorptive capacity, OCLs are efficient phagocytes (4–6). They can process, present, cross-present antigens, and activate T cells (7–9). They also produce cytokines and immunomodulatory factors driving immune response towards inflammation or tolerance (7, 10–13), and thus are important players in immunologic homeostasis in the bone marrow niche and in the whole interactome of osteoimmunological processes. Moreover, similar to Mφs, OCLs exhibit phenotypic and functional heterogeneity (7, 12, 14) and can arise from different precursors depending on the bone microenvironment and the stimuli they receive (15). These findings that both bone phagocytes contribute to immune responses not only emphasize the functional similarities between OCLs and Mφs, but also demonstrate that the role of OCLs extends far beyond their bone resorption function. Thus, the field of osteoimmunology has gained significant interest through the identification of Mϕ populations from different origin and polarization states within the bone, together with the increased appreciation of OCL diversity. This review aims to highlight how both cell types similarly and differentially participate in maintenance of bone metabolism and the bone marrow environment during steady state conditions, and represent possible target in case of hormonal, inflammatory or traumatic disruption of their niche. We discuss the latest reports deciphering their analogies in cell progenitors, phagocytosis, and immune function, as well as functional and characteristic differences in certain aspects such as bone resorption or fusion under physiological conditions. In regard to related pathophysiological conditions, we focus on 3 main reasons for bone disorders of diverse origin importantly mediated and/or resolved by Mϕ and OCLs; in detail, we will discuss primary Osteoporosis as a consequence of age and hormonal disorder, fracture resolution and healing as traumatic impact in need of intervention of bone marrow phagocytes, and finally, Rheumatoid Arthritis, as an auto-inflammatory disease affecting the joints and bones.

## Macrophages: characteristics and functions

Mφ populations have been identified in all tissues where they exert organ-specific functions and have specific transcriptomic signatures, suggesting a major influence of the local tissue-specific environment on their genome expression and functions (16), as e.g., Mϕ located in different tissues rely on differential transcriptional programs as indicated by dependence on certain transcription factors. This high plasticity of Mφ still leaves many unanswered questions, particularly with regard to environmental, organ-specific or pathological influences, the respective outcomes and functions of this diversity, and appropriate targets to intervene with specific Mφ subsets in the context of pathologies (17).

Similar to OCLs and their progenitors, tissue resident Mφs express Macrophage Colony-Stimulating Factor receptor (CSF1R) and are dependent on M-CSF for their maintenance. CSF1R expression gradually increases during Mφ differentiation from common myeloid progenitors (CMP) and granulocyte/Mφ progenitors (GMP) (18). In the bone marrow (BM), M-CSF produced by endothelial cell provides a niche that regulates Mφ/OCL/dendritic cell progenitors (MODP), and monocytes (MNs) (19). Thus, CSF1R drives the differentiation, survival, proliferation and chemotaxis of Mφ and is used as a Mφ and MN marker (18). Of note, *Csf1r*-deficient mice not only show a drastic decrease in Mφ but also in OCL number, which is associated with an osteopetrotic phenotype (20).

Of special interest for this review are Mφs residing in the bone, where they account for 15-20% of bone marrow resident immune cells (21). Osteal Mφs (OsteoMACs) represent a specific subset of Mφs residing in bone lining tissues that are phenotypically characterized as F4/80^+^CD169^+^ Mac-2^−/low^ cells, while non-skeletal tissue Mφs are identified as F4/80^+^Mac‐2^high^ cells (22). Resident bone Mφs are present in close contact to osteoblasts and are involved in bone formation, maintenance and repair by osteoblasts (22, 23). Furthermore, under osteoporotic conditions characterized by increased bone catabolism, OsteoMACs spatially join resorbing OCLs and assist in the clearance of resorption by-products (24). These studies highlighted the indispensable position of OsteoMACs in both bone modeling and remodeling. OsteoMACs are involved in the recruitment of osteoblast precursors and provide osteoblasts with coupling-like factors (as TGFβ and Ephrin B) necessary for bone formation (25), while in turn, they are supported by osteoblast-derived M-CSF (26). Nevertheless, the exact origin and functions of OsteoMACs, their crosstalk with OCLs and their precursors, and their contribution to bone homeostasis require further investigation.

## Osteoclasts: characteristics and functions

Similar to Mφs, OCLs derive from myeloid progenitor cells; but contrasting to Mφs, they are physiologically multinucleated and arise as a result of fusion events of various precursor cells that either reside in or are actively recruited to the BM. Osteoclasts are specific to bone and are characterized by their ability to resorb bone. Bone remodeling involves complex and tightly regulated interactions between OCLs, osteoblasts and their environment (3). However, in pathological conditions when this homeostasis becomes unbalanced, such as chronic inflammatory diseases, OCLs can become a major pathogenic player leading to skeletal tissue damage and osteolysis.

OCL differentiation, fusion and activation are triggered by stimulation of Receptor Activator of NFκB (RANK), which is expressed on progenitor cells and early-stage OCLs, with its corresponding ligand, RANK-L (27, 28) produced by various cell types, including osteoblasts, osteocytes, mesenchymal stromal cells (MSCs) and their adipocyte lineage progeny, as well as certain immune cells, such as activated CD4^+^ T cells (28, 29). As stated above, M-CSF is another indispensable factor for OCL differentiation (30). In addition to RANK-L and M-CSF, there is a variety of other regulators, such as cytokines and hormones, that stimulate or inhibit OCL differentiation, making it a tightly regulated and complex process (31).

Classical OCL differentiation induced by RANK-L and M-CSF activates signaling cascades that cumulate in transcription of genes indispensable for osteoclastogenesis and resorption, e.g., *Acp5*, *Ctsk* or *Calcr*. Activation of RANK induces signaling via the adaptor protein TRAF6 that subsequently targets pathways such as NFκB, AP1/JNK/Jun/c-Fos, p38, ERK and Src/PI3K/AKT axes, which all induce *NFATc1*, the master gene of osteoclastogenesis (32). OCL differentiation also engages the co-stimulatory pathway involving Immunoreceptor Tyrosine-based Activation Motif (ITAM)-containing receptors associated to Fc gamma receptors (FcγR) or DAP12, and OSCAR or TREM2, respectively. This signaling cascade involves Syk signaling to induce NFATc1 via PLCγ and Ca2^+^ signaling (33–37). Of note, these receptors also play an important role in the differentiation and function of other cells from the innate immune system including Mφs and DCs.

Mature OCLs are the sole cell type capable to resorb mineralized bone matrix, which requires the coaction of tightly regulated and complex cellular processes. Attachment to the bone surface via adhesion molecules to form podosomes is indispensable to form sealing zones around the resorption lacunae (38, 39), and to maintain OCL mobility during this process which dictates a resorption mode (40). These lacunae resemble giant extracellular lysosomes, since subsequent massive acidification is required to degrade the inorganic matrix, while secretion of proteinases degrades the organic compounds. Finally, resorption products are endo- and transcytosed to the apical OCL domain to be released into the extracellular environment (41). This constitutes an important mechanism to the coupling of bone resorption to formation and thus bone remodeling, since the secretion of resorption factors impacts on osteoblast recruitment, maturation and activity (3, 42).

Although OCLs are cells of the myeloid lineage and display characteristics of innate immune cells, their potential role as immune cells has long been neglected. However, recent findings in the field of osteoimmunology have shown that besides their bone resorption capacity, OCLs are also true innate phagocytes and APCs (15). As such, they are able to actively shape the immune environment and immune responses. Studies demonstrated their capacity for phagocytosis, antigen uptake and presentation, as well as their efficient cytokine production in response to physiological and pathological stimuli (6–8, 43). This important finding that OCLs are not only bone resorbing cells but also, similarly to Mφs, involved in immune responses sheds new light on OCLs and makes them interesting targets to prevent bone diseases (15).

## Common characteristics and differences among macrophages and osteoclasts

### Common origin of bone phagocytes

**Figure 1:**
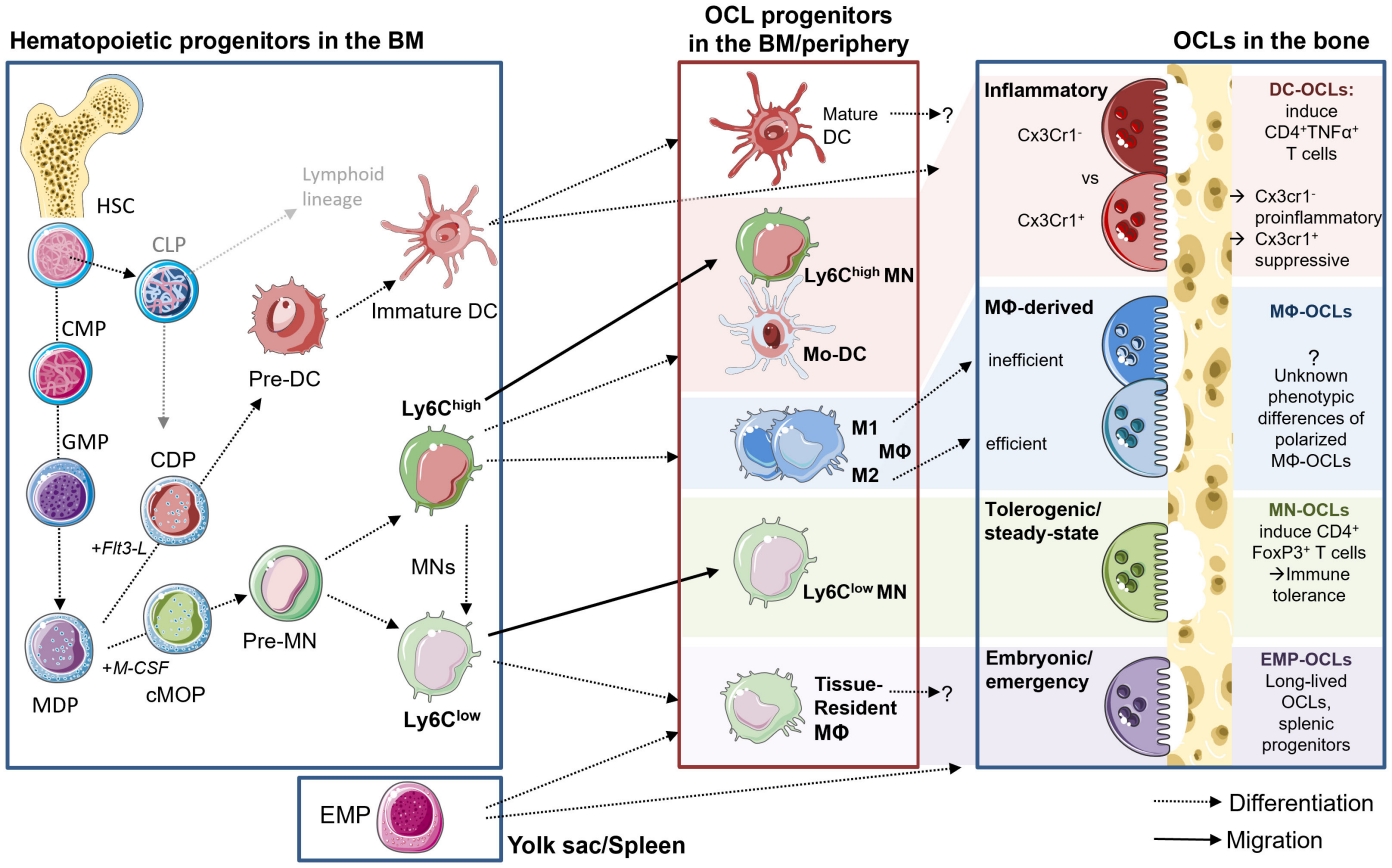
Diversity of OCLs and their progenitors. Left side: hematopoietic lineage and progenitors in the BM. HSCs give rise to MODPs via intermediate CMP and GMP states. M-CSF stimulation induced pre-MNs and further Ly6C^high^ and Ly6C^low^ MNs, while Flt3-L induces the DC lineage. Middle box: myeloid cells can leave the BM and enter into tissues via the circulation for maturation and polarization or they reside and differentiate within the BM and possibly give rise to OCLs. Upon appropriate stimulation with M-CSF and RANK-L, these myeloid cells can fuse and give rise to phenotypically diverse OCLs. Macrophage-derived DCs and Ly6C^high^ MNs give rise to predominantly pro-inflammatory OCLs, while Ly6C^low^ MNs generate tolerogenic OCLs. During embryogenesis and fracture repair, EMP-derived progenitors from the spleen give rise to OCLs. M1 polarized Mφ have been described as inefficient for OCL formation, while M2 possess a higher potential for osteoclastogenesis, but the resulting OCLs phenotype has not yet been described particularly.

Until recently, Mφs and OCLs were considered to be strictly derived from BM progenitors and to arise from a common MN-Mφ/OCL progenitor (cMoP) downstream of GMP and MODP, both in mouse and in human (44–46) (**Figure 1**). These progenitor cells give rise to blood MNs that can reach tissues and differentiate into Mφs (47, 48). Similarly, transfer of blood monocytic cells (Kit^−^Ly6C^+^) into osteopetrotic Ctsk^-/-^ mice defective in OCL activity was shown to rescue bone resorption, demonstrating the contribution of these cells to osteoclastogenesis (49).

However, more recent reports established that the origin of Mφs and OCLs is more complex. In particular, embryonic precursors seed subsets of tissue Mφs that persist into adulthood completely independent of blood MN replenishment (50). In mice, embryonic Mφs derive from erythro-myeloid progenitors (EMP) in the yolk sac (around E7 of embryonic development) or from fetal liver progenitors (around E16) (51). Postnatally and throughout adult life, embryonic Mφs persist through proliferation in order to sustain a constant pool of tissue-resident Mφs in most organs (51). Importantly, tissue resident Mφs co-exist with postnatally generated MN-derived Mφs and participate in organ homeostasis during health and disease. Of note, Mφ ontogeny could influence their functions (52) (**Figure 1**).

Bone-resident OsteoMACs have a trophic role in bone formation and mineralization, while MN-derived bone Mφs show pro-inflammatory properties. Both are involved in osteoclastogenesis and OCL formation (53–55) and both participate in osteoporosis development associated with chronic inflammatory diseases or infection due to their production and secretion of pro-osteoclastogenic factors such as IL-6, TNFα or IFNβ (26). In the case of bone injury in mice, deletion of CCR2 reduces MN infiltration that subsequently affects the generation of MN-derived inflammatory Mφs, and severely impairs the function of OCLs, leading to delayed fracture healing (56). Collectively, these data suggest that there are at least two different Mφ subsets co-existing in the BM that both carry out selective functions and roles in bone physiology as well as during bone healing after injuries. Nevertheless, the relative contribution of MN-derived and embryonically-derived Mφs to bone repair is still to be determined and the interaction of OsteoMACs with OCLs remains to be fully established. In particular, deciphering the precise transcriptomic signature of the diverse BM Mφs needs to be further investigated by single cell approaches to better understand BM Mφ diversity which could help to precisely determine their functions.

While the progenitors of Mφs have been widely discussed in the literature, the origin of OCLs has long remained elusive and continues to evolve, mainly because OCLs arise not only from hematopoietic progenitors but also from the fusion of mature myeloid cells (15) (**Figure 1**). The myeloid origin of OCLs has been identified 40 years ago *in vivo* using blood MNs labeled with ^3^H-thymidine in mice treated with 1α-OH Vitamin D to stimulate osteoclastogenesis. In recipient mice, radiolabeled OCLs were detected showing the osteoclastogenic potential of MNs, but they represented only a small proportion of total OCLs (57, 58). Later, different analyses demonstrated that OCLs share a common MODP with Mφs (45, 46). However, OCLs originating from BM HSC-derived progenitors appears to be involved only after birth and in adults. In contrast, during embryonic development and in neonatal bones, OCLs differentiate from embryonic yolk sac-derived EMPs as described for Mφs, as confirmed by fate mapping experiments in transgenic mice baring irreversible fluorescent marker (TdTomato or YFP) expression in Csf1r^+^ cells and their progenies (14, 49). At birth, 80% of bone OCLs originate from EMP-derived Mφs, but this proportion gradually decreases with time as EMP-derived OCLs are replaced by HSC-derived OCLs (14, 49). However, a small fraction of EMP-derived Cx3Cr1^+^ Mφs persist in the spleen and contribute as precursors of a small pool of OCLs throughout adulthood (14). In addition, they can migrate from the spleen to the bone upon injury, where they contribute to bone healing (14). Therefore, as described for Mφs, adult long bones contain co-existing, yet very distinct OCL populations originating from either Cx3Cr1^+^ EMP or HSC-derived progenitors (14, 49). However, the respective functions of these populations, in particular beyond bone resorption, remain unexplored (**Figure 1**).

As discussed above, OCL arise not only from myeloid progenitors but also from differentiation/fusion of mature monocytic cells, i.e. MNs (MNs), Mφs and dendritic cells (DCs), making this issue highly complex (14, 15, 49, 53, 59, 60). As described in the next sections, this differentiation depends on the persisting environment, and the phenotype of these cells also impacts the functionality of the mature OCL in mouse and in human (7, 12, 15, 61). These mature myeloid cells are themselves highly heterogeneous and come in various polarization states (**Figure 1**). Typically, blood MNs are divided into two major subsets. First, circulating classical/inflammatory MNs are recruited to tissues in a cytokine-dependent manner, where they differentiate into Mφs and DCs responsible to migrate and resolve local inflammation (62–64). Inflammatory MNs are characterized by the markers CD14^high^ CD16^neg^ in human and CD11b^+^ CD115^+^ Ly6C^high^ in mouse (65). Second, non-classical MNs (human CD14^+^ CD16^high^ and mouse CD11b^+^ CD115^+^ Ly6C^low^) are the housekeepers of endothelial borders by constantly fulfilling a patrolling function in order to quickly detect and react to injury or inflammation/infection (64). Both MN subsets are involved in inflammatory processes due to their cytokine production, however, their osteoclastogenic potential differs both in human and murine models. Especially during conditions of increased bone resorption or even destruction, inflammatory MNs were shown to differentiate into mature OCLs much more efficiently than the non-classical MNs (66–68).

On the other hand, DCs have been also shown to be efficient OCL progenitors (15) (**Figure 1**). Initially, the differentiation of OCLs from DCs has been demonstrated *in vitro* starting from human blood MN-derived DCs, which was shown to be increased in presence of synovial fluid from arthritic patients (60). *In vitro*, murine OCLs can be generated from ex vivo isolated splenic CD11c^+^ MHCII^+^ cDCs (69). Interestingly, the CD4^-^CD8^-^ cDCs possess a much higher OCL differentiation capacity than other DC subsets. cDCs even maintain this capacity after their maturation induced by stimulation with CpG or LPS, indicating that also mature cDCs are still able to give rise to OCLs (69). Murine OCLs have also been generated *in vitro* from purified CD11c^+^ DCs obtained with GM-CSF/IL-4 stimulation of BM cells (7, 70) or from Flt3-L-induced DCs (71). DC-to-OCL differentiation also occurs *in vivo*, as initially demonstrated in osteopetrotic *oc/oc* mice deficient for *Tcirg1* which is required for bone resorption. Transfer in newborn *oc/oc* mice of splenic cDC from normal mice rescued the bone phenotype through their differentiation into functional OCLs (69). Of note, this differentiation requires the presence of Th17 cells, the proportion of which is increased in the BM of oc/oc mice (69). The differentiation of DCs into OCLs has always been primarily associated with chronic inflammation or with the presence of Th17 cells (7, 60, 69, 72–74).

To add further complexity, short-term stimuli of the microenvironment can directly impact the polarization state of the respective progenitors, which is not a “either-or” static condition but rather a flexible, fluent spectrum that can quickly adapt to the environment. In regards to osteoclastogenesis, the influences acting on the progenitors before initiation and during fusion directly determine the functional outcome of the respective OCLs (7, 12, 15, 61). Unfortunately, it is unknown whether these different types of progenitor cells select a specific type of cell as fusion partner or whether a mixture of multiple diverse progenitors can form an OCL. In addition, OCLs not only undergo cell fusion but also cell fission, as reported *in vitro* (75) and *in vivo* (76). *In vivo*, OCL fission is regulated by RANK-L and generates so-called osteomorphs, a cell type characterized by a gene signature distinct from both OCLs and Mφs, which is able to recycle by fusing again with OCLs. This new mechanism can contribute to a rapid and energy-efficient generation of OCLs (76) as well as to the long live span of OCLs that has been recently demonstrated *in vivo* (49). Gaining insight into these processes could help to answer open questions in regards to OCL diversity in physiological versus pathological conditions, taking into account the variation in the proportions of these cells in the BM as well as their situation-dependent recruitment from the periphery. This will be essential to better understand pathological bone destruction and to unravel novel targets to interfere at multiple levels.

### Fusion: a common feature between Mφs and OCLs

Linking progenitor diversity, the impact of the surrounding environment and cellular function of both Mφ and OCLs together, one process is importantly involved in this notion: fusion of mononucleated cells to form multinucleated syncytia. Physiologically, this is a rare phenomenon that can only be found in certain tissues such as the muscle, the placenta, or OCLs in the bone. Otherwise, multinucleation is associated with pathologic conditions such as reactions to foreign material or chronic inflammation such as granulomas, viral infections or cancer and metastasis (77–80). Under these conditions, Mφs have the potential to fuse and to give rise to multinucleated giant cells (MGCs) (39, 77). Depending on the environmental state, these MGCs can display differences in their appearance and can be further distinguished based on histological observations. Contrasting with OCLs, these cells do not resorb bone. For the sake of simplicity, we will refer here to all Mφ polykaryons as MGCs and recommend this review about the different subtypes and their pathological associations (77). In contrast to MGC differentiation, osteoclastogenesis is a constantly occurring event that is indispensable for the maintenance of bone homeostasis.

In general, cell fusion is a multi-step process with different checkpoints (81). A prerequisite for Mφ fusion is the emergence of a fusion-competent state of the cell, which is characterized by the induction of specific molecules that often follow a time-dependent expression pattern. Fusion involves the principle of attachment of a “founder cell” to a “follower cell”, meaning that both fusion partners do not necessarily have to express the required molecules (82). Appropriate exogenous and endogenous stimuli (cytokines, fusogens) induce chemo-attraction and attachment and ultimately membrane fusion of partner cells (39, 81).

A couple of studies suggest that OCL progenitors preferentially fuse with phenotypically divergent partner cells in terms of different states of differentiation and mobility in a very dynamic process over time (83). Moreover, the authors also describe differences in the choice of fusion partners, i.e. 2 mononucleated progenitors, 1 mononucleated and 1 multinucleated, 2 multinucleated OCLs as well as the initial surface attachment, which might also be interesting as indicators for OCL diversity as well.

Regardless of their common origin, OCLs and Mφ polykaryons differ considerably, which might be related to the particular local environment in which they develop. In regards to cytokines, as noted above, RANK-L is the driving cytokine for osteoclastogenesis, but has no reported effect on MGC development. Conversely, MGCs are mainly induced by the type 2 cytokines IL-4 and IL-13 (84, 85), that both inhibit osteoclast formation (86, 87). Furthermore, OCL differentiation requires M-CSF (30), whereas MGCs are induced in the presence of either M-CSF or GM-CSF (88).

Despite these differences, the mechanisms of fusion have been shown to share similarities between OCLs and Mφs (89) and some molecules involved in Mφ fusion are known OCL markers (summarized in **Table 1**). In addition to the conventional RANK-L axis, the IgG-like triggering receptor expressed by myeloid cells 2 (TREM2) and its adaptor protein DNAX activating protein of 12kD (DAP12) induce an important co-stimulatory signaling pathway in the initiation of osteoclastogenesis (36). Deletion of either partners reduced OCL differentiation, multinucleation, and function (34, 90). Interestingly, this Ig-like receptor-associated pathway seems to be differentially involved in OCL differentiation depending on the origin of OCLs from dendritic or monocytic cells (61). Concurrently, TREM2 and DAP12 are equally involved in MGCs development, as their deletion completely abrogated Mφ priming for fusion competence (91). Downstream of IL-4 activation of Mφs, TREM2/DAP12 signaling via STAT6 can lead to transcription and induction of further fusion mediators, e.g., E-cadherin and DC-specific transmembrane protein (DC-STAMP), which are important mediators of fusion and cell-cell attachment (91, 92). Inhibition and knockout of E-cadherin have been shown to severely impair MGC development and also reduce OCL formation (92–94). DC-STAMP is involved in the maturation of myeloid cells and was originally described on DCs (95, 96). However, it is also expressed on IL-4 stimulated Mφs as well as OCL progenitors and turns out to be indispensable for osteoclastogenesis and cell fusion (97–99). During OCL differentiation, this could be explained by a corresponding regulation of its expression via the RANK-RANK-L/c-Fos/Nfatc1 signaling axis (98). BM cells deficient in DC-STAMP failed to fuse into multinucleated OCLs despite M-CSF and RANK-L stimulation (99). Interestingly, however, the remaining mononucleated TRAP^+^ cells still expressed other OCL markers and resorbed bone to a certain extent, provoking a mild osteopetrotic phenotype in the DC-STAMP-deficient mice *in vivo* (99). This supports the hypothesis that increased cell fusion correlates with increased phagocytic capacity in OCLs (100). In humans, transcript variants of DC-STAMP are associated with Paget´s disease of bone, which is characterized by hyper-nucleated OCLs, emphasizing a direct functional implication of this protein in the fusion processes (101). Moreover, expression levels of DC-STAMP on human OCL progenitors are correlated to OCL nuclei number *in vitro* (fusion potential) and activity markers *in vivo*, both of which directly related to the resorptive activity *in vitro* (102). DC-STAMP expression levels were reported to be positively associated to the donor´s age and menopausal status, regulated through an epigenetic mechanism (102).

**Table 1:** Examples of fusogens and receptor-ligand interactions involved in cell fusion.

Molecule	Function	Expressed on/at	Reference
TREM2/DAP12	Phagocytosis Cell fusion Osteoclastogenesis Inflammation through cytokine stimulation	Myeloid cells	(36, 90, 91, 243)
E-cadherin	Ca2+- dependent adhesion molecule Transmembrane glycoprotein	Many cell types, especially at adherens junctions	(92–94, 244)
DC-STAMP	Myeloid maturation and fusion Osteoclastogenesis	DCs, Mφ, OCLs and their progenitors	(95, 97, 99, 101, 116, 245, 246)
CD47	Immunoglobulin-like protein Don´t eat me signal to prevent phagocytosis Adhesive protein	Ubiquitous	(103, 105, 106, 247)
MFR/SIRPα	Mφ fusion Immunoglobulin superfamily Receptor of CD47 Regulation of phagocytosis	Mainly myeloid cells	(103, 105, 248)
CD200	Membrane glycoprotein Immunosuppression Produced by peripheral tissues	Many cell types including Mφs and OCL progenitors just before fusion	(107)
CD200R	Receptor for CD200 Myeloid downregulatory signal	Myeloid cells	(107)
CD44	Cell-cell interaction Adhesion, migration	Mφ about to adhere and fuse	(111–113, 249)
OC-STAMP	Master fusogen of Mφ and OCL precursors	Mφ, OCL progenitors (as DC-STAMP)	(114–116, 149)
MCP1/CCL2	MN homing/recruitment Fusion processes	Many cell types	(119–121, 250, 251)
CD9, CD81 (CD63)	Migration, motility, invasion Differentiation Attachment, fusion Interaction with integrins	Very broad	(117, 127–130, 132)

Various members of the immunoglobulin-like superfamily have been shown to be associated with both Mφ and OCL progenitor fusion. Mφ fusion receptor (MFR/SIRPα) and its ligand CD47 are prominent examples of respective ligand-receptors interactions expressed in the early stage of fusion (103). MFR is mainly expressed on myeloid cells including Mφs, cDCs and OCLs, whereas CD47 is ubiquitously expressed as a self-signal in order to prevent phagocytosis by Mφs (103, 104). During fusion, CD47 allows cells to get in close proximity and absence of either of the interacting partners was shown to reduce OCL differentiation both *in vitro* and *in vivo* (105), but CD47 seems to be more important for fusion of mononucleated rather than multinucleated cells (106).

Another member of the immunoglobulin-like family, CD200R, is expressed on myeloid cells and interacts with CD200 which is not expressed in myeloid cells except on Mφs just before fusion (107). CD200 expression is also stimulated by RANK-L in pre-OCLs and its expression is maintained in mature OCLs (10, 107). CD200 is produced in various tissue in order to regulate Mφ activity and is known as an immunomodulatory/immunosuppressive molecule associated with cancer, infection and autoimmune diseases (108–110). Cui et al. showed that mice lacking CD200 (or its receptor CD200R) display delayed and reduced OCL differentiation *in vitro*, which could be reversed by the addition of soluble CD200. Consistent with these *in vitro* observations, CD200^-/-^ mice have increased bone mass, indicating that the CD200-CD200R-axis plays a crucial role in Mφ fusion and osteoclastogenesis (107).

The glycoprotein CD44 is expressed on Mφs and OCLs, and associated with cell-cell interaction, adhesion and migration, and is important for fusion events. As for CD200, CD44 expression increases in Mφs prior to fusion and is required for OCL multinucleation *in vitro* (111, 112). However, the role of CD44 in osteoclastogenesis appears more complex and seems to depend on the environment. Indeed, *in vivo*, bone parameters of CD44-deficient mice are similar to those of control mice (111, 113) but they exhibit differences after stimulation as reported in a hindlimb-unloading mouse model in which CD44 deficiency impairs OCL differentiation *in vitro* and *in vivo* (111). Moreover, *in vitro* the effects of CD44 on osteoclastogenesis depend on the substrate on which the OCLs are differentiating (113). Importantly, it has been suggested that an increase in CD44 expression during fusion is required to provide free CD44 molecules to favor multinucleated, which is abrogated in the presence of CD44 ligands (e.g., hyaluronic acid, chondroitin sulfate or osteopontin) (112).

OC-STAMP, structurally similar to DC-STAMP but described to be specific for OCLs, is also induced upon RANK-L stimulation and responsible for OCL precursor fusion (114). Analysis of OC-STAMP deficient mice demonstrated its role as a master fusogen for Mφs and OCL precursors, as its deletion leads to abrogated multinucleation of both OCLs and MGCs *in vitro* and *in vivo* (115, 116). However, in contrast to DC-STAMP-deficient mice (99), OC-STAMP -/- mice neither display differences in their bone architecture nor in the level of resorption markers in the serum, indicating that despite being mononucleated, OCLs in these mice are functionally active (115, 116). However, in a murine model of ligature-induced periodontitis, OCL activity is lower in OC-STAMP-/- mice than controls, suggesting that despite not being involved in physiological bone resorption, OC-STAMP may play a role in inflammatory bone loss (117). Interestingly, the ligands for DC- and OC-STAMP have not been identified so far, but as their structure displays some similarities with chemokine receptors, the MN chemo-attractant protein 1 (MCP1/CCL2) has been suggested as a potential ligand (118). CCL2 is a chemokine required for MN homing and recruitment to peripheral tissues as well as for fusion of both Mφs and OCL progenitors. Mice deficient for CCL2 or its receptor CCR2 displayed reduced MGC and OCL numbers, despite normal MN/Mφ migration and infiltration (119). Moreover, other studies shed light on a possible autocrine feed-forward mechanism of OCL-secreted CCL2 to induce further fusion through its receptor CCR2, which in turn is induced by RANK-L stimulation (120, 121).

The tetraspanin family participates in a huge variety of cellular functions by influencing membrane organization (122). Tight interaction with integrins (themselves being important players of fusion events and probably regulated in a time- and cell-type-dependent manner during fusion enables their influence on cell motility, attachment and fusion (39, 123). Two members of this family seem to be of importance in Mφ fusion: CD9 and CD81. Despite their reported fusogenic effect in muscle cells, sperm-oocyte fusion and infection-induced syncytia (124–126), CD9 and CD81 appear as rather negative regulators of MGC and OCL fusion. Anti-CD9 and CD81 neutralizing antibodies significantly increase MGC and OCL development (127–129). CD9/CD81-deficient mice have increased OCL formation and lower bone mass than controls (129). However, CD81 was suggested to exert its negative effects via modulation of CD9 rather than by itself (128). In contrast to these findings, Yi et al. found that neutralization of CD9 reduced osteoclastogenesis, suggesting that CD9 positively regulates OCL differentiation (130). CD9 is highly expressed on OCLs in inflammatory conditions such as osteoporosis induce by ovariectomy or collagen-induced arthritis in mice (131). As for DC- and OC-STAMP, Ishii et al. also reported that CD9 expression increases in OCLs at the time of fusion upon RANK-L stimulation and this increase is mediated by OC-STAMP (117, 132). Therefore, further investigations are required to gain a better understanding of the impact of tetraspanins in Mφ and OCL fusion. Summed up, mechanisms of fusion are still not well enough understood in order to be targeted to combat pathophysiologic Mϕ fusion or accelerated osteoclastogenesis.

### Cellular heterogeneity and immune function

Since Mφ and OCLs belong to the myeloid family of innate immune cells, they both share characteristic features of this family, including phagocytosis, antigen presentation capacity as well as the ability to induce adaptive immunity.

#### Phagocytosis and efferocytosis

Mφs and OCLs share a very efficient phagocytic ability. BM Mφs locally participate in the phagocytosis of pathogens, apoptotic and necrotic cells in different contexts such as injury or cancer, as well as in removing residual by-products of osteoclastic bone resorption (24, 133). They can also phagocytose wear debris derived from prostheses (134). CD169^+^ BM Mφs are involved in the clearance of apoptotic cells and of the nuclei eliminated from erythrocytes during erythropoiesis (135). However, little is known regarding the specific ability of OsteoMACs to present antigens, to express MHCII and costimulatory molecules and to activate T cells. In any case, they can actively participate in pro-inflammatory immune responses through other mechanisms as well, for example by the expression of pathogen recognition receptors (PRRs) like Toll-like receptors (TLRs) to recognize pathogen- or damage-associated molecular patterns (PAMPs or DAMPs) (136). In the case of microbial infections, bacterial lipopolysaccharide (LPS) triggers TNFα secretion by OsteoMACs through the activation of TLR-4/CD14 complex (25). Secreted TNFα subsequently promotes IFNγ production by Th1 and NK cells, in turn capable to further activate Mφs and promote a pro-inflammatory immune response directed against the microbial infection (137). These data suggest that bone Mφs have, as classical Mφs, a phagocytic and sentinel function to prevent bone infection and excessive inflammation.

Interestingly, fusion has been suspected to increase the phagocytic capacity of MGCs compared to mononucleated Mφs. MGCs originating from Mφs under IL-4 stimulation are specialized in the phagocytosis of very large particles and opsonized particles for which they are much more efficient than mononuclear Mφs (92, 100). MGCs have also been shown to degrade very large particles extracellularly after their tight attachment to the particle with the formation of a sealing zone-like structure reminiscent of that formed by OCLs for bone degradation (138). However, and as discussed above, MGC do not form ruffled border and are not efficient for bone resorption (138).

Of note, OCLs are also efficient phagocytes for the uptake of large particles of different types (6, 100, 139) including calcium-phosphate crystals (4). They engulf bone remnants for the purpose of bone degradation through clathrin-mediated endocytosis (140, 141). They are also involved in bacterial phagocytosis and in efferocytosis (5, 142–145). In particular, several studies indicate that OCLs phagocytose dying bone cells including osteocytes and chondrocytes. Harre et al. reported that OCLs express multiple proteins involved in the engulfment of apoptotic cells, to a similar or even higher extend than Mφs and DCs (144). *In vivo* evidence revealed that they perform phagocytosis under physiological conditions (142). Furthermore, OCLs and their progenitors sense necrotic osteocytes thanks to their expression of Mincle, the Mφ-inducible C-type lectin, which stimulates osteoclastogenesis and participates in pathological bone loss (146). Efferocytosis is essential for the clearance of dying cells, and defects in this process lead to chronic inflammatory and autoimmune diseases. Because osteocytes and chondrocytes are surrounded by abundant bone and cartilage matrices, respectively, it is more difficult for classical phagocytes such as Mφs to reach and eliminate them. Thus, the capacity of OCLs to perform efferocytosis is likely to be essential for the maintenance of the bone/BM integrity (144). Interestingly, expression of PRR and c-type lectin receptors by OCLs depends on their cell origin from monocytic cells or dendritic cells (61). As this origin is governed by the bone marrow environment (7, 60, 69), this suggests that the activity of OCLs can differ depending on their context.

Linking phagocytic capacity to the expression of PRRs represents a hallmark of professional antigen presenting cells; before antigen phagocytosis, presence of PAMPs potently activate PRRs, which acts as the molecular link between myeloid antigen ingestion, processing and subsequent presentation via MHCII that enables bridging to adaptive immunity, a feature that both Mφ and OCLs share.

#### Antigen presentation and T cell activation

Just as Mφs, OCLs are innate immune cells that uptake, process, present and cross-present antigens (7–9). They constitutively express major histocompatibility complexes (MHC) class I and II as well as costimulatory molecules such as CD80 and CD86 (7, 8, 60), and are therefore able to shape the immune response by activating naïve CD4^+^ and CD8^+^ T cells in an antigen dependent manner. This was first demonstrated for CD8^+^ T cells that can be activated by antigen cross-presentation by steady-state, physiologic OCLs and subsequently polarized into CD8^+^ FoxP3^+^ regulatory T (Treg) cells (8). Concurrently, the same was also shown for CD4^+^ T cells that are similarly primed towards CD4^+^ Treg cells by steady-state OCLs (7, 9). Thus, steady-state OCLs have a tolerogenic capacity by inducing regulatory T cells (t-OCLs, tolerogenic OCLs) that is likely to represent an efficient mechanism to avoid auto-immune reactions against self-antigens released by the OCLs from the bone matrix during resorption. Moreover, because Treg cells are potent inhibitors of osteoclastogenesis, it may also represent a feedback loop controlling OCL number and activity, which has been confirmed for OCL-activated CD8^+^ Treg cells (147).

However, similar to Mφs and other cells of the myeloid lineage, OCLs exhibit functional heterogeneity. Recent findings indicate the existence of distinct OCL subsets that arise from different progenitor cells depending on the microenvironmental stimuli and polarization state of mononuclear precursor cells before the onset and during the course of fusion (15, 61). Thus, pro- or anti-inflammatory subsets of Mφs and MNs give rise to functionally distinct OCL subsets (7, 15, 61, 66, 68, 109). As stated above, tolerogenic OCLs arise during steady-state conditions (t-OCLs) and from BM MNs (MN-OCLs) (7, 61). However, in inflammatory conditions such as inflammatory bowel diseases (IBD) (7), rheumatoid arthritis (RA) (60), osteoporosis (12, 61) and osteopetrosis (69), CD11c^+^ DCs or inflammatory CD11b^+^ Ly6C^high^ MNs can also give rise to OCLs (DC-OCLs). Although DC-OCLs are also resorbing bone, they exhibit drastic differences in their immune function. Contrasting with t-OCLs that induce Treg cells, DC-OCLs promote the development of TNFα-producing pro-inflammatory T cells involved in systemic inflammatory processes, therefore termed inflammatory OCLs (i-OCLs) (7, 12). These studies further emphasize the functional similarity between Mφs and OCLs, ranging from tolerance to inflammation. But these functional differences also require to be phenotypically distinguishable in order to properly identify, differentiate and target for therapeutic applications.

### Cellular markers and new potential targets

**Figure 2:**
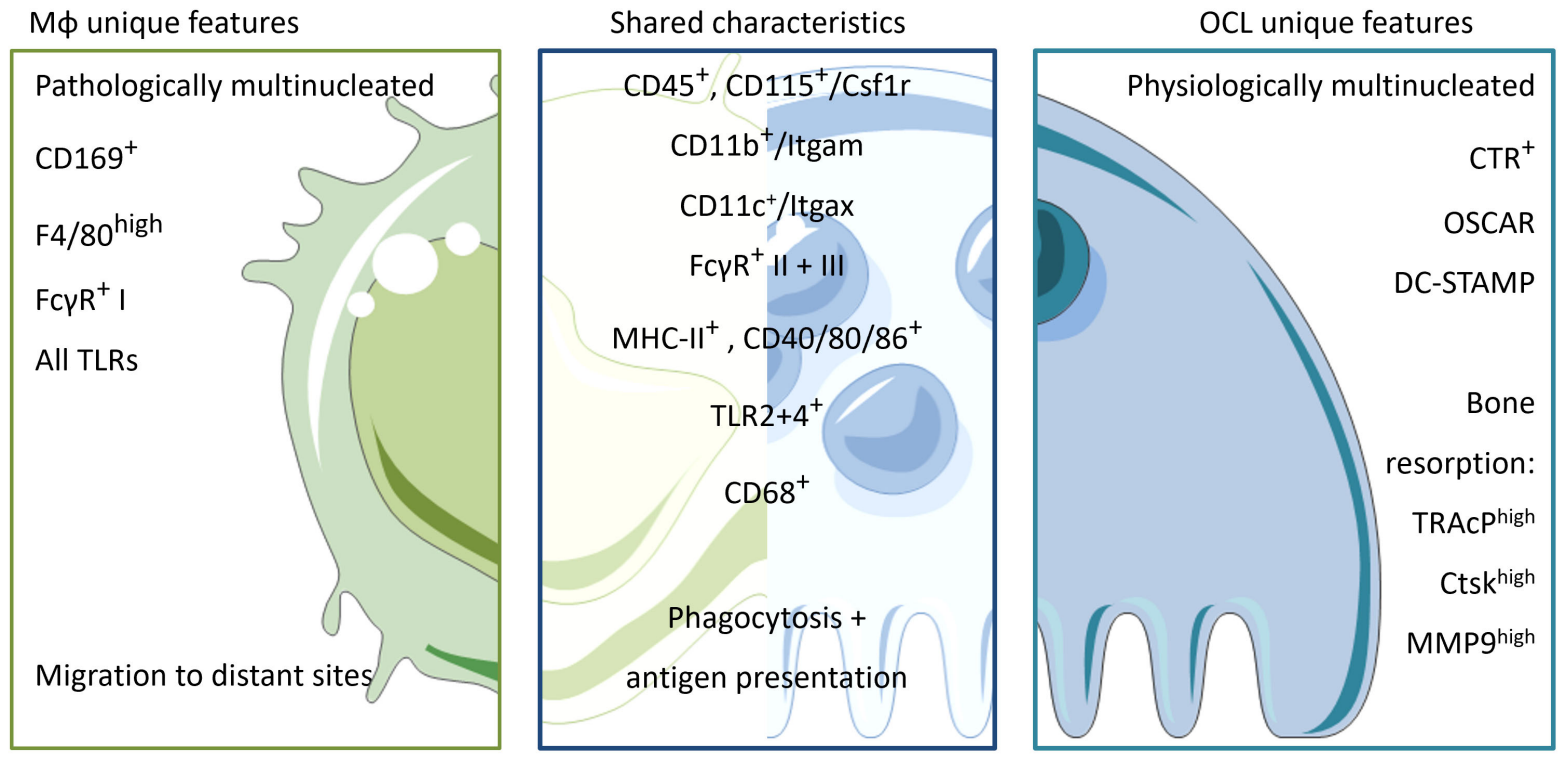
Unique characteristics and shared features between Mφs and OCLs. Macrophages and OCLs share a lot of similarities. Both arise from the hematopoietic lineage and depend on M-CSF maintenance (CD45+CD115+). They share common expression of integrins (CD11b+, CD11c+) and an innate immune function markers involved in phagocytosis and antigen recognition/presentation such as FcγR I-III, MHC-II and co-stimulatory molecules CD40/80/86. Macrophages are physiologically mononucleated and able to be attracted ad migrate to distant sites in response to infection/inflammation. They express all the repertoire of TLRs to sense their environment, and can be identified by the expression of pan-macrophage marker F4/80 and CD169 for bone-marrow macrophages. In contrast, OCLs are physiologically multinucleated, able to resorb bone and are therefore localized in the bone compartment only. To distinguish from macrophages, OCLs express CTR, OSCAR and DC-STAMP on their surface as well as enzymes involved in the resorptive process e.g. Trap, Ctsk and MMP9.

Mφs and OCLs share an abundance of characteristic markers and common functions due to their myeloid origin. Therefore, identification of specific markers *in vitro* is challenging. Furthermore, the shared environmental influences of bone resident Mφs and OCLs can lead to a large overlap of markers and functions (**Figure 2**, **Table 1**). Nevertheless, there are some major differences. For instance, TRAcP is a suitable marker expressed by mature OCLs but it has been reported to be expressed only in very few and specific Mφ subsets (148, 149). Recently, a method for analysis of mixed cultures of multinucleated OCLs and their progenitors has been developed that uses nuclear staining and doublet exclusion strategy to focus on pure OCL populations (150). This approach will help to uncover more differences to distinguish OCLs from other myeloid cells during live cell analysis. Functionally, mature OCLs can be discriminated by culture on biomimetic bone surfaces in order to follow matrix degradation capacity, which is a unique feature of OCLs but not of Mφs.

As mentioned above, OsteoMACs express TLRs to participate in pro-inflammatory immune responses during microbial infections. Moreover, OCLs are also known to express TLRs. Takami et al. showed that stimulation of these TLRs by their respective ligands act as potent inhibitors of OCL differentiation (151) (**Figure 2**). Other research groups, however, showed opposite effects between freshly isolated progenitors and committed precursors induced by RANK-L pretreatment or combined effects of impacting environmental cues (152). A recent review discusses these contrasting observations regarding TLR/PRR stimulation and its effects on osteoclastogenesis, and elaborates on how this might be explained by considering differences in OCL differentiation stages (153). Nevertheless, these discrepancies might also be related to OCL heterogeneity. Indeed, combining transcriptomic and flow cytometry analysis of mature OCLs, we recently showed that TLR2 expression (along with other PRRs) differs dramatically between tolerogenic MN-OCLs and inflammatory DC-OCLs (61). Moreover, stimulation of OCLs with PRR agonists strongly and very selectively affects the differentiation of OCLs associated with inflammation, whereas physiological OCLs are much less affected. Thus, targeting PRRs could represent a novel and interesting pharmacological intervention in osteoporotic pathologies (61).

Closing the loop of TLR activation and subsequent cytokine production by Mφs, proinflammatory cytokines are a known factor influencing OCL precursors and osteoclastogenesis (59, 154, 155). TNFα, which exerts its stimulatory effect through different mechanisms, can be used to illustrate these influences. First, TNFα indirectly increases RANK-L and M-CSF production by mesenchymal lineage cells in the bone (156, 157). As RANK is a member of the TNF receptor superfamily, it is not surprising that TNFα is strongly affecting osteoclastic bone resorption, osteoblastic bone formation and consequently also bone remodeling. Second, as it is the case with several cytokines, TNFα can act directly on OCL progenitors and osteoclastogenesis. This is particularly the case in inflammatory diseases associated with bone destruction (59, 158). TNFα in the absence of RANK-L is not sufficient to induce fusion of OCL progenitors (159, 160). Nevertheless, it is capable of increasing differentiation efficiency alone or in cooperation with other proinflammatory cytokines such as IL-1α, IL-1β or IL-17 (155, 161, 162). Moreover, TNFα also induces a dramatic expansion of OCL progenitors in the circulation, which migrate to sites of inflammation where they fuse into OCLs (163). Interestingly, Ohori et al. have recently shown that TNFα-induced acceleration of bone destruction can be reversed by simultaneous administration of IL-33, which prevents TNFα-induced IkB phosphorylation and subsequent NF-κB nuclear translocation, thereby inhibiting osteoclastogenesis *in vitro* and *in vivo* (164). In synergy with these new insights into communication networks between the different players in the inflammatory circuits and the findings on signaling cytokines, numerous novel possibilities for pharmacological interventions are emerging and might lead to interesting developments in the near future.

Preceding investigations on functional divergences of Mφs and OCLs, it is essential to identify specific markers that allow clear distinction and separation between these cells present in the BM, for example, by deciphering specific and unique characteristics for each cell type (**Figure 2**, **Table 2**). Unfortunately, bone resident Mφs are still poorly characterized, and the identification of markers allowing to distinguish them from DCs, MNs, and especially from OCLs, is urgently needed. The first marker described for Mφs was F4/80 (165). However, it is now known that this marker is expressed by different Mφ subtypes but is also found on MNs, CD11b^+^ DCs and eosinophils, for instance (16, 166). Moreover, F4/80 expression is dependent on the cytokine environment and is therefore not a stable marker (167).To date, Mφ populations can be characterized by the co-expression of MerTK and FCγR-I (CD64) (16). These findings are consistent with the functional implication of FcγR-I, -II and -III (CD64, 32, 16, respectively) in phagocytic uptake and antigen presentation (168, 169). In line, OCLs are known to express FcγR-II and III, but this expression is higher in i-OCLs (61). Therefore, differential labelling of Mφs and OCLs using cell surface markers remains challenging, especially considering the wide heterogeneity of OCL progenitor cells including DCs, MNs, BM and osteal Mφs. Excepted for very few markers such as CD169 expressed by Osteomacs but not OCLs (22), the vast majority of markers identified for OCLs are shared with their myeloid progenitors (e.g. Csf1r, CD11b, CD11c, CD68 (170), Cx3cr1 (7, 12), TLRs (61) (**Table 2**). However, combining these markers with parameters for size and multinucleation still allows for accurate and differential analysis (61, 150) (**Figure 2**). Furthermore, being able to clearly define the precise functional differences of the various Mφ populations present in bone (generally identified as CD68^+^ F4/80^+^ TRAP) (21, 25), will allow a better understanding of bone physiology. Finally, in-depth knowledge of how these Mφ subpopulations interact with each other and with other skeletal cell types, as well as a deeper understanding of how they influence bone homeostasis and osteoclastogenesis in pathological conditions, will unravel novel targets and treatment options for bone diseases.

**Table 2:** Major markers common to Mφs and OCLs .

Molecule	Expressed by	Function	Over-lap	References
CSF1R	Myeloid cells	M-CSF receptor	MNs, Mφs, OCLs	(252–254)
F4/80	Mφs	Adhesion, induction of CD8+ Tregs	Blood cells	(16, 165, 166)
FCγRI-III	Phagocytes	Immunoglobulins: immune function, phagocytosis	All phago-cytic cells	(16, 168, 169)
CD68	Mφs	Involved in phagocytosis, apoptotic clearance, cell-cell/pathogen contact, homing	MNs, OCLs	(25, 170)
Cx3cr1	Mφs, some i-OCLs	Fractalkine receptor		(7, 12, 240)
TLR2/4	Immune cells	Starting immunological signaling cascades to resolve inflammation	Mφs, MNs, DCs, i-OCLs	(61, 151, 152, 255)
RANK	OCL progenitors, early OCLs	Receptor of osteoclastogenesis	MNs, Mφs, DCs	(256–259)
TRAP	Mature OCLs, Mφs, and DCs	Bone resorption processes, Ag processing clearance of the pathogen	MNs, Mφs, DCs	(260, 261)

## Perspectives in pathological contexts: osteoporosis, rheumatoid arthritis and bone fractures

**Figure 3:**
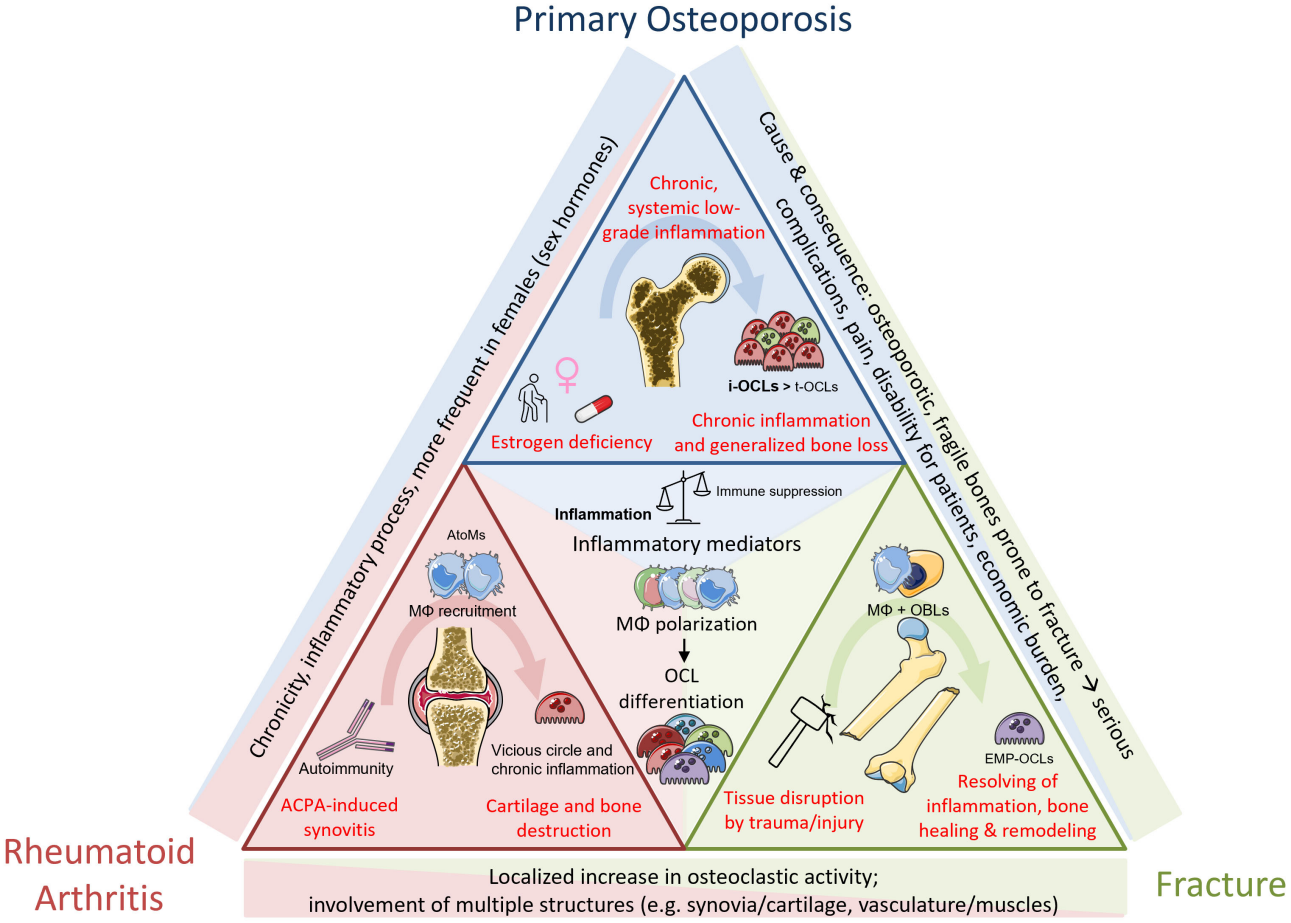
Common and differential mechanisms between pathological mechanisms in osteoporosis, rheumatoid arthritis and fracture repair. Osteoporosis is primarily associated with gender, age and medication/other conditions. Systemic low-grad inflammation leads to increase in osteoclastogenesis and generalized bone loss. RA is an autoimmune disease caused by ACPA, mediating local joint inflammation and generalized bone destruction. Fractures happen upon traumatic injuries and are divided not several phases including different cell types. Bone healing after callus formation is mediated by OCL-dependent remodeling mechanisms. Despite the different origins, the inflammatory component as well as increased progenitor fusion to form OCLs are a shared mechanism partaking in all these conditions.

Because of their similarity and essential role in the BM, OCLs and Mφs are both key players in the development of various bone pathologies. In this last part, we will discuss perspectives in pathological contexts of bone. There is of course more to consider, however, we will limit the focus to mechanisms involved in osteoporosis (OP), rheumatoid arthritis (RA) and fracture healing of long bones (Fx) (**Figure 3**).

### Osteoporosis

The most widespread pathology affecting the bone is osteoporosis, a skeletal disorder characterized by a systemic loss of bone mass, leading to architectural deterioration and ultimately to brittle and fragile bones, and increased risk of fracture. The underlying causes are multiple but are very much associated with gender (post-menopausal lack of estrogen) and aging (decrease in physical activity, metabolic disorders, additional medical conditions, pharmacological treatments, lifestyle, etc.). It can also occur as a consequence of chronic diseases (**Figure 3**). Osteoporosis is diagnosed by measure of the bone mineral density by dual-energy x-ray absorptiometry (DXA). A DXA t-score from -1 to -2.5 upon is defined as osteopenia, a decrease in bone mass, which can evolve to osteoporosis when t-score is <-2.5 (171). Both conditions are mainly due to an imbalance between osteoblastic bone formation and osteoclastic bone resorption, the latter one exceeding the physiological rate, thereby impacting on the coupling mechanisms between these two cell types. A recent meta-analysis calculated the general world-wide prevalence of osteoporosis as 18.3% (23.1% in women and 11.7% in men, respectively) (172). Unfortunately, the predictions for annual fragility fractures in Europe indicate an increase of around 25% within the next decade, mainly due to an increase in the elderly population (173). Currently approved therapies are predominantly anti-resorptive by targeting OCLs. Bisphosphonates, the most prescribed anti-osteoporotic drugs, are structurally modified phosphate molecules that integrate into inorganic matrix. They prevent the OCL from proper attachment or induce apoptosis (174, 175). However, all bisphosphonates provoke side effects, especially after long-term treatment, which can lead to rare but severe adverse events: they affect the coupling of osteoblasts to OCLs, thereby also bone formation rates. This disbalance eventually weakens the bone (176) or can possibly further induce medication-related osteonecrosis of the jaw (177), a severe condition which is thought to be at least partly induced by a direct effect of bisphosphonates on Mφ polarization (178).

More recently, the monoclonal anti-RANK-L antibody Denosumab was developed to treat bone loss by pharmacological inhibition of osteoclastogenesis (179). Despite its great efficiency in preventing OCLs to form, discontinued therapy can lead to a subsequent acceleration of bone destruction in patients (180). A recent study hypothesized a mechanisms for OCLs to self-maintain upon RANK-L withdrawal by fission of intermediates called osteomorphs from mature OCLs, that recycle and refuse with one another or different OCLs once the appropriate stimuli return (76). This novel picture could explain an increase of osteoclastogenesis and osteoclastic bone destruction after Denosumab withdrawal. Therefore, alternative targets to treat pathological bone loss are needed to improve therapeutic options.

Postmenopausal osteoporosis is also defined as a chronic low-grade inflammatory condition. Estrogen withdrawal has multiple direct and indirect effects on the bone. They are reported to directly inhibit osteoclastic bone resorption via its receptor ERα and to induce apoptosis of OCLs (181). Furthermore, they are well-known to exert an important impact on different Mφs regardless their location, as, for instance, estrogen withdrawal also increases the risk for atherosclerosis (182), impairs skin balance and wound healing (183) and affects immune cells (184) such as bone-resident Mφs.

Physiological levels of estrogen were shown to block osteoclastogenesis from M2 Mφs via ERα (185), despite M2 possessing a higher OCL differentiation potential than M1 Mφs (186). In the ovariectomized (OVX) mouse model, lack of estrogen altered the M1/M2 ratio of Mφs in the bone. While M2 Mφs differentiate into OCLs, M1 Mφs do not and persist in the BM (185, 187). This sustains a vicious circle of maintained production of pro-inflammatory M1 cytokines whilst the regulatory factors coming from M2 Mφs are absent, thus shaping osteoclastogenesis to feed-forward the inflammatory condition (12, 15, 154).

In the BM, multiple players shape the environment by secreting cytokines capable to regulate Mφ differentiation, activity and polarization, including T cells and myeloid cells; as a consequence, OCL differentiation and phenotype are equally affected, which can accumulate in osteoporosis (188, 189). Pro-inflammatory/Th1 cytokines (e.g. IL-1, IL-6, IFNγ, GM-CSF and TNFα) induce M1 Mφs (190) that in turn also produce the same kind of cytokines, resulting in a feed-forward exacerbation of inflammation. Some of these pro-inflammatory cytokines consequently increase osteoclastogenesis (154), while others are reported to decrease efficiency of OCL differentiation (191). The same increase of pro-inflammatory cytokines was found in postmenopausal women, inducing systemic inflammation and increased MN and lymphocyte populations (192).

In addition to their effects on Mφ polarization and resulting OCLs, T cells in osteoporosis also directly affect osteoclastogenesis. Importantly, Th17 CD4^+^ cells and their signature cytokine IL-17 are reported to be the most potent pro-osteoclastic T cell population (193), to contribute to inflammatory bone loss *in vivo* (59, 194) and to induce the formation of inflammatory OCLs (7). On the contrary, anti-inflammatory/Th2 mediators (mainly IL-4 and IL-13) induce M2 Mφs that maintain a tolerogenic environment by production of IL-10 and IL-4, which both are reported to inhibit osteoclastogenesis (86, 195). Reciprocally, as mentioned before, mature OCLs also actively shape the T cell composition and thus the pro-inflammatory environment in the BM; in contrast to healthy, steady-state conditions, the osteoporotic BM is enriched in TNFα^+^ CD4^+^ cells, which are induced by inflammatory OCLs. Moreover, in osteoporotic conditions, Cx3cr1^+^ OCLs associated to pathology are increased, suggesting a causal participation to the screw in T cell populations (15, 61). To date, it is still controversial to definitely conclude about the pathogenic/pathologic impact of the presence or absence of specific cytokines and reports might be conflicting. Therefore, one must carefully consider the diversity of the various cytokines involved, investigate their fine-tuning mechanisms as well as their respective abundances and possible dual modes of action. More research is needed to unravel the specific composition during physiological versus pathological circumstances to allow specific targeting as a therapeutic option.

Importantly, as OP is a chronic inflammatory disease, other organs are affected during the onset and progression of the disease, e.g., the intestinal compartment. OP is associated with changes in the gut microbiome (196–198), which weakens the intestinal epithelium barriers and increases gut permeability (194, 199), a process that is mainly mediated via ERβ signaling (200). This stimulates gut inflammation and leads to the activation and amplification of inflammatory Th17 cells. These cells, as well as microbial products, are capable to disseminate to the bone compartment where they exacerbate inflammation and induce the emergence of pro-inflammatory OCLs (7, 59, 61, 194, 201). Reducing gut inflammation with bacterial probiotics limits osteoclastogenesis and bone loss in osteoporotic mice (202, 203). Interestingly, we showed recently that treatment of OVX mice with a probiotic yeast improved gut leakiness, restored normal levels of bacterial short chain fatty acids (SCFA) and reduced the proportion of inflammatory T cells in the BM, leading to a diminished proportion of inflammatory OCLs. Therefore, specific targeting of inflammatory osteoclastogenesis limits the bone loss induced by ovariectomy (61).

In summary, OP is a multifaceted disease that is induced, influenced, and aggravated by multiple pathways. Hormonal changes, gut inflammation, cytokine composition and other inflammatory diseases can exert detrimental effects on the progression of pathological bone loss. Interestingly, increasing evidence points towards a generalized inflammatory state during aging, called “inflammaging” (204) due to overproduction of pro-inflammatory cytokines by Mφs, which could shed light on the development of bone-related pathologies.

### Rheumatoid arthritis

RA is an autoimmune disease affecting the joints but also characterized by extra-articular manifestations. The global prevalence is 0.46% (205) and it represents a major cause of sick leaves, hospitalization, physical handicap and early retirement, with an about 2-fold increased risk of mortality compared to the general population (206). RA represents an increasing global socioeconomic burden with an increasing number of patients worldwide (206). RA starts as a local inflammation of the synovial tissue of various joints that becomes chronic over time, causing cartilage erosions and bone destruction at juxta positioned sites that eventually culminate in pain, disability and risk of fracture (207, 208) (**Figure 3**). Bone destruction can become systemic leading to secondary osteoporosis in about 50% of the patients (209). Autoimmunity against citrullinated proteins (due to the presence of anti-citrullinated protein antibodies, ACPA) is one of the main risk factors in RA, in particular associated with an increased risk to develop bone erosions (**Figure 3**). ACPA levels are used as a serological marker to detect RA even before the onset of clinical manifestation as well as to predict disease progression. Bone erosion can occur very early in ACPA positive patients even before the first articular manifestation (210). Clinically, the major therapeutic options are disease modifying anti-rheumatic drugs (DMARDs, e.g. methotrexate) in combination or not with other drugs that regulate inflammation (such as cytokine inhibitors) or interfere with osteoclastogenesis (denosumab, bisphosphonates) (211). TNFα inhibition efficiently reduces local joint inflammation and interferes with OCL differentiation, thereby providing a beneficial effect on bone in those patients that did not respond to other treatments (212, 213). As discussed earlier, TNFα is reported to positively stimulate osteoclastogenesis via direct or indirect mechanisms. It has been shown to even be sufficient in inducing osteoclastogenesis in combination with IL-6 in a RANK-KO model (214). Despite the variety of possible combinations, more than 4 out of 10 patients do not respond to methotrexate, including the ones that develop severe adverse effects such as gastrointestinal events and liver toxicity (215). Thus, new therapeutics are also urgently needed to reach non-responders and to reduce therapy-associated problems.

Mφs are reported to be the main drivers of RA development and expansion and are found highly diverse in the inflamed synovium (53, 216–218). Under steady-state conditions, self-renewing tissue resident Mφ subsets of embryonic origin populate the synovium and carry out very specialized yet distinct functions to maintain the integrity of the synovial capsule (217, 218). Contrasting, in murine models of RA, it was found that these populations shift in both morphology and phenotype (217), while there was also infiltration of MN-derived Mφs that participate in inflammation by differentiating into pro-inflammatory Mφs or OCLs (217–219). In the serum-transfer model of arthritis (STA), which is independent of the adaptive immune system and resolves after around 12 days, non-classical Ly6C^low^ blood MNs that gave rise to MHCII^+^ inflammatory Mφs initiated the inflammatory phase, while tissue-resident MHCII^-^ Mφs were involved in STA resolution. This latter phase was also characterized by a switch from M1 Mφs during initiation towards M2 resolving phenotype (218). Additionally, non-classical Ly6C^low^ MNs were localized at sites of bone erosion together with OCLs while classical Ly6C^high^ MNs were absent (220). However, the data on OCL differentiation potential from these different MN subsets is conflicting and results are still under debate (68, 220). In a different model of murine RA, the collagen-induced arthritis (CIA), another study reported Ly6C^high^ classical MNs as driver population for RA, both by the production of TNFα and the downregulation of the immune-regulatory ant anti-osteoclastogenic microRNA miR-146a (221). While specific targeting of Ly6C^high^ MNs with miR-146a containing liposomes did not alter the arthritic score, it abrogated bone loss in these animals (221).

In line with these preclinical studies, the amount of articular Mφ infiltration is also used as a direct measure of disease activity and remission in humans patients (222). Moreover, transcriptomic data suggested that Mφ diversity in murine RA might resemble the human disease situation (223). Recent investigations further confirm that distinct populations might be involved in different phases of the disease and remission, by possessing diverse capacities to progress or resolve inflammation (224). However, the classical M1/M2 categorization might not be valid in this setting, since polarization states are thought to be mixed in terms of markers and functions (225). Hasegawa et al. recently described a BM-derived Mφ population (referred to as AtoMs) in the joint of mice with CIA as well as in human RA patients with a particularly high potential to differentiate into OCLs and to induce bone erosions (53). This population is characterized by the expression of FoxM1, a transcription factor they also suggest as a target (53). More markers for Mφ subsets associated to RA are being discovered, and chemokines are importantly involved in the recruitment of potential OCL progenitors to the inflamed joint. As an example, inhibition of the chemokine receptor 2 (CCL2/CCR2) axis (by pharmacological depletion or genetic tools) has shown to be efficient in preventing circulating CCR2^hi^ MN populations from infiltrating the joints in murine RA, which did not alter the inflammatory score but abrogated OCL differentiation and bone erosions (68, 218, 226). Additionally, the CCL21/CCR7 axis was also described to be highly involved in RA progression (227). Increased levels of CCL21 in the synovial fluid of patients attract CCR7^+^ monocytic cells to the inflamed joint, where they polarize towards M1-like Mφs (227). Via IL-6 and IL-23 production, these Mφs induce Th17 polarization of naïve T cells, which possess a high osteoclastogenic potential and therefore partake in exacerbation of RA by inducing bone erosion (193, 228). In patients, the IL-17 level can be elevated in the serum and synovial fluid, which also correlates with disease severity (229). Combining different cytokines might also display an interesting option to catch 2 birds with 1 stone, e.g. shaping Mφ polarization at the same time as Th induction. Importantly, both directions of Mφ-T cell crosstalk again play a role that should not be neglected.

### Fracture healing of long bones

In 2019, the global number of fracture cases reached around 178 million and is more common in men than in women as well as in the elderly population (230). Regarding the number of cases described each year and the resulting economic burden in terms of costs for hospitalization, rehabilitation or potential permanent handicaps, a better understanding of the underlying molecular mechanisms in fracture repair along with the implication of diverse immune cell populations could improve the healing process through potential pharmacological modulation of their actions.

The fracture repair process is initiated by BM immune cells that are able to remove debris and dead cells, which allows the creation of an endochondral callus in the beginning, that is followed by the remodeling phase. Fractures come together with a disruption of vasculature and soft tissues at the site of the trauma. This vasculature disruption allows the formation of a hematoma where platelets, MNs and neutrophils are recruited from the blood vessels (**Figure 3**). Aggregating platelets form a thrombus that induces immune cells (including neutrophils, MNs and Mφs) to secrete various chemokines and cytokines (231). In this process, Ly6C^hi^ Cx3cr1^low^ CCR2^hi^ inflammatory MNs are recruited by chemokines secreted by bone resident immune cells such as resident Mφs/OsteoMACs and MSCs at the site of injury. When this recruitment of MN is impaired, the number of Mφs strongly decreases (25). Moreover, this MN recruitment leads to an increase of IL-1^+^, IL-6^+^ and TNFα^+^ (232) and dysregulation of cytokine secretion seems to impair also bone healing (233). These MNs will differentiate into Mφs under the control of the CCL2-CCR2 axis (234). The role of OsteoMACs in this early stage of MN recruitment has also been investigated using Mafia (Mφ Fas-induced apoptosis) mice. Depletion of OsteoMACs in MAFIA mice impaired bone healing after fracture (133). If the depletion occurred at the time of the surgery, there was no callus formation (235), demonstrating that the newly recruited, MN-derived inflammatory Mφs are important in the initial steps of fracture repair. Indeed, CCR2^-/-^ mice, which display impaired MN recruitment, show a decrease of Mφ infiltration at the site of the fracture 3 days after the injury. This phenomenon was shown to impair vascularization, highlighting the role of Mφs in this first important phase of trauma resolution (56).

In the next step, the formation of a fibrocartilaginous callus takes place around day 7 post-injury. In the soft callus, the new cartilage matrix could be observed as soon as day 7 after fracture, and was found to align with the fracture gap by day 14 post-fracture (236). In this process of endochondral bone formation, there is a proliferation of mesenchymal cells and their progenitors, which are committed to an osteochondral progenitor lineage during this period. However, the role of Mφs in this process remains largely unclear. CCR2-deficient mice exhibit a smaller callus at day 7 post-fracture, but there was no impact on the total volume of new bone or cartilage at this point (56). Depletion of OsteoMACs and MN-derived Mφs at day 5 also reduced the size of the callus during the anabolic phase (235). Therefore, it seems that OsteoMACs are important in the early stage of the fracture repair in order to secrete chemokines attracting MNs that give rise to Mφs, which allows the re-vascularization of the thrombus. Both populations of Mφs seem to be importantly involved in the formation of fibrocartilaginous callus to sustain mesenchymal cell proliferation and differentiation.

The transformation of a soft fibrocartilaginous callus into the hard boney callus follows this process. This hard boney callus is then remodeled to form the new bone structure. Mφs, OsteoMACs and OCLs are implicated in this late remodeling process. In particular, OCLs are crucial in this remodeling process of the hard callus, to allow the formation of a bone structure with similar dimensions to the pre-fractured bone (237). OCLs are thought to be less important in cartilage remodeling, as their depletion during the early cartilage remodeling process by OPG did not affect callus remodeling but instead delayed fracture repair (238). However, some studies have also shown that OCLs are able to degrade cartilage (239). Interestingly, during bone healing, the spleen represents a source of a new wave of hematopoiesis. Indeed, after a fracture, in Cx3cr1 ^CreERT2^; R26^tdTomato^ mice labeled at E9.5, tdTomato^+^ cells were present in the red pulp of the spleen (14). When the spleen of these mice is removed, the number of tdTomato^+^TRAP^+^ OCLs decreases at the site of injury, suggesting a contribution of splenic Mφs to the formation of OCLs after bone injury (14). However, it seems that the contribution of circulating MNs to the OCL pool is a minor event under homeostatic condition but is restricted to fracture events, as shown with parabiotic experiments (240). Additionally, intravenous injection of OCL progenitors into mice has proven incorporation of these cells into mature OCLs only during fracture repair, not in control mice (240). Mφs are also involved in this part of the process but the exact contribution between OsteoMACs and MN-derived Mφs remains unclear. Although OsteoMACs and Mφs play more important roles in the previous part of intramembranous ossification, they do not seem to be crucial for this process. Overall, Mφs act preferentially during the first steps of fracture repair in order to clean and create a new matrix for osteoblastic bone formation and subsequent remodeling by OCLs to ultimately obtain a new bone structure.

## Conclusion

Osteoimmunology is an interdisciplinary and increasingly evolving field that describes the interplay between immune and bone cells and the role of OCLs and Mφs in this field is crucial. Major research advances in recent years have shown that Mφs and OCLs play an essential role in the BM microenvironment and regulate the balance of bone remodeling and bone resorption. Current research approaches focus mainly on the unidirectional action of Mφs on OCLs during polarization. However, OCLs are also involved in the production of different cytokines that significantly affect Mφs and the BM microenvironment. In addition to their common origin from hematopoietic progenitor cells, OCLs and Mφs share a wealth of similarities regarding their characteristics and function. In particular, with respect to their immune function, both cell types share common signaling pathways and, like other cells from the myeloid lineage, both cells exhibit high cellular plasticity. The finding that both OCLs and Mφs contribute to immune responses demonstrates that the role of OCLs extends far beyond their bone resorption activity and expanded the scope of osteoimmunology.

Because of their similarities and essential role in the BM, both OCLs and Mφs are instrumental in the pathogenesis of a variety of skeletal diseases. These disorders mainly involve overproduction of inflammatory cytokines by Mφs and increased OCL differentiation leading to imbalanced bone resorption. It is therefore critical to determine the precise triggers and underlying molecular signaling pathways to better understand the contribution of each cell type in order to gain deeper insights into the pathologies associated with OCLs and Mφs.

In the clinical point of view, to date, most therapeutic options for the aforementioned bone-related pathologies have been disease-modifying drugs that regulate inflammation or interfere with osteoclastogenesis. This is similar to clinical practice during chronic inflammatory diseases, as chosen biologicals interfere with certain mediating cytokines but provoke severe side effects due to their indispensable role in fighting e.g. infection or cancer (241). There is a lack of approaches that specifically target the interaction of different cells in the bone microenvironment; in part, this can be explained by the relatively difficult access to human samples to study steady-state and pathological conditions, as well as important challenges in preclinical models to establish e.g., intravital imaging, fate mapping experiments inside the bone marrow niche, problematic ex vivo isolation of viable cells etc. Despite, it is of uttermost importance to further determine cellular phenotypic changes, exact triggers and signaling pathways involved in various pathologies to more accurately comprehend the contribution of OCLs and Mφs, and to finally develop targeted approaches for the clinics. As described before, preclinical data is available in this regard, and is still attracting increasing interest. As such, the identification of targetable surface markers specific for certain OCL subsets identified options to specifically reduce OCLs associated to inflammation in the osteoporotic background (61); other reports describe distinct transcriptional programs of progenitor cells as main drivers of bone loss in RA, highlighting a possible point of interference with this specific progenitor subpopulation (53); the development of nanoparticles to deliver targeted inducers of apoptosis of both Mφ and OCLs in the arthritic joints to combat inflammation (242). In summary, the identification of specific targets to interfere with the emergence of Mφ as drivers of inflammatory conditions directly or as OCL progenitors, and in the same way osteoclastogenesis or originating pro-inflammatory subsets, will be key to develop targeted therapeutic strategies and to reduce side effects in treatment options for patients. In this outlook, recent technological advances such as single-cell RNA sequencing will help to further improve the molecular understanding of OCLs and Mφs during health and disease. In combination with spatial imaging and transcriptomic, they will unravel important insights into specific location, cellular interactions, spatial proximities and communication networks between Mφs and OCLs. This will give important insights on their phenotype and function directly in their native environment, as well as their role in the BM microenvironment maintenance in health and dysregulation during disease to enable the identification of novel pharmacological targets addressing their pathological association.
